# Outcomes associated with total neoadjuvant therapy with non-operative intent for rectal adenocarcinoma

**DOI:** 10.3389/fonc.2024.1374360

**Published:** 2024-07-29

**Authors:** Ebenezer Asare, Emily Venner, Hanna Batchelor, Jason Sanders, Paul Kunk, Traci Hedrick, Sook Hoang, Matthew Reilley, Tri Le, Charles Friel, Einsley-Marie Janowski

**Affiliations:** ^1^ Department of Radiation Oncology, University of Virginia, Charlottesville, VA, United States; ^2^ University of Virginia School of Medicine, Charlottesville, VA, United States; ^3^ Department of Hematology/Oncology, University of Virginia, Charlottesville, VA, United States; ^4^ Department of Colorectal Surgery, University of Virginia, Charlottesville, VA, United States

**Keywords:** rectal adenocarcinoma, total neoadjuvant therapy, radiation therapy, colostomy, watch and wait

## Abstract

**Purpose/objective(s):**

To evaluate rates of clinical complete response (cCR), surgery-free survival, permanent ostomy-free survival, and factors associated with these outcomes in patients treated with total neoadjuvant therapy (TNT) with intent for non-operative management of rectal adenocarcinoma.

**Methods:**

A retrospective review was conducted of patients treated with TNT for stage II-IV rectal adenocarcinoma (n=45) at our institution between 2013 – 2022 with curative intent. All patients received radiation with concurrent capecitabine and additional chemotherapy, either prior to or following chemoradiation (CRT), with intent for non-operative management. Response rates were determined based on post-treatment MRI and endoscopy. Kaplan-Meier method was utilized to estimate the 1- and 2-year surgery- and permanent ostomy-free survivals. Cox regression was used to evaluate associations between surgery- and permanent ostomy-free survivals and various factors of interest, including patient and tumor characteristics and clinical response. Chi-squared analysis compared rates of cCR and surgery by sequence of TNT modality and cell count ratios.

**Results:**

Of the 45 patients treated with TNT, most patients had low-lying rectal tumors with a median distance of 4.1 cm from the anal verge (range, 0.0 – 12.0). Overall, 64.4% (n=29) achieved cCR after TNT. 13 patients (28.9%) underwent surgical resection following TNT, 12 of whom had incomplete response and one who elected to undergo surgery after reaching cCR. At median follow up of 32.0 months (range, 7.1 – 86.1), 22.2% (n=10) of patients had a permanent colostomy, with only 2 of these completed for tumor regrowth after cCR. At one and two years, respectively, surgery-free survival was 77.3% and 66.2%, and permanent ostomy-free survival was 90.9% and 78.2%. Rates of cCR were higher in patients who received CRT first compared to those who received chemotherapy first (72.2% vs. 33.3%, *p*=0.029) and rates of surgery were also lower in patients who received CRT first compared to those who received chemotherapy first (19.4% vs. 66.7%, p=0.005). On Cox regression model, cCR on 6 month post-CRT endoscopy was associated with surgery-free survival (p=0.006) and permanent ostomy-free survival (*p*=0.033). Clinical response at earlier follow up points did not predict surgery- nor permanent ostomy-free survival.

**Conclusion:**

These results support evidence that TNT may be a non-surgical option for select patients with rectal adenocarcinoma who desire organ preservation. In this investigation at a single institution, the treatment response on 6-month post-CRT endoscopy was the best predictor of surgery- and permanent ostomy-free survival, which are outcomes that are important to patient quality of life. CRT followed by consolidation chemotherapy was associated with higher rates of cCR and lower rates of surgery compared to those treated with induction chemotherapy.

## Introduction

The American Cancer Society estimates that there will be 46,050 new rectal cancer diagnoses in 2023 (1). Over the past two decades, there has been a shifting paradigm in the standard treatment for rectal adenocarcinoma. Treatment for locally advanced rectal cancer is multimodal and traditionally consists of neoadjuvant chemoradiation (CRT) or short-course radiation followed by surgical resection followed by adjuvant chemotherapy. The standard surgical resection, known as a total mesorectal excision (TME), has two main approaches depending on the location and extent of the tumor: low anterior resection (LAR) which may involve the creation of a reversible ostomy, and abdominoperineal resection (APR), which involves removing the entire distal rectum and anus including sphincters and results in a permanent colostomy (2). Surgical resection of low-lying rectal cancers often requires an APR because of the location in the pelvis and proximity of the tumor to the anal sphincter, which significantly impacts patients’ quality of life (3).

Treatment strategies have shifted toward total neoadjuvant therapy (TNT) in which all chemotherapy and radiation is completed prior to surgery rather than surgery followed by adjuvant chemotherapy. In 2004, the German Rectal Trial established that preoperative CRT improved local control and was associated with reduced toxicity compared to postoperative CRT (4). The RAPIDO trial further contributed to evidence that in addition to preoperative CRT, neoadjuvant chemotherapy was likely more efficacious than adjuvant chemotherapy (5). The more recent publication on locoregional failure from the RAPIDO trial provides some concerns on the efficacy of short course radiation for maintained local control in their high risk group of patients, but continues to show improvement in distant metastasis when both radiation and chemotherapy are completed neoadjuvantly (6). More recently, the STELLAR trial demonstrated non-inferiority of short-course radiation therapy and neoadjuvant chemotherapy (TNT) compared to neoadjuvant CRT for the primary endpoint of 3-year disease-free survival, but showed hints of improved overall survival for a TNT approach (7).

The Organ Preservation in Patients with Rectal Adenocarcinoma (OPRA) trial assessed disease free survival in patients with stage II-III disease treated with a non-operative intention with either preoperative CRT followed by consolidation chemotherapy versus induction chemotherapy followed by CRT (8). Patients treated with a selective watch-and-wait strategy based on tumor response had similar disease-free survival and TME-free survival compared to historical controls treated with TNT. Patients who underwent CRT followed by consolidation chemotherapy had higher rates of organ preservation than the induction chemotherapy arm. This trial supported selective non-operative management of patients with rectal adenocarcinoma following TNT, with comparable outcomes to historical controls.

There is growing evidence that selective non-operative management of patients with locally advanced rectal cancer can reduce surgery-related morbidity and permanent ostomy creation while preserving disease-free survival rates (9, 10). For patients with locally advanced low-lying rectal cancer, non-operative management with TNT alone has been increasingly utilized. The International Watch & Wait Database was established to compile a large-scale representation of patients treated with this strategy to track outcomes of the treatment. Findings from this multicenter registry demonstrate high rates of disease-free survival after cCR (9, 10). In our single institution analysis, we identify outcomes in patients with locally advanced rectal cancer who were treated with a selective non-operative approach based on tumor response to TNT to contribute to the body of evidence supporting a selective watch-and-wait strategy in rectal cancer. We also aim to correlate putative biomarkers of treatment response including lymphopenia and the platelet-lymphocyte ratio (PLR) in our patient population, which have been previously reported to be prognostic in colorectal cancer (11–13).

## Materials and methods

A retrospective review was conducted of patients treated with TNT for stage II-IVA rectal adenocarcinoma at our institution between 2013 and 2022. 124 patients in total received chemoradiation over this time period, however, patients with low to mid lying rectal cancer were selected for non-operative intent received TNT to avoid a morbid surgery. All patients received TNT consisting of radiation therapy (XRT) with concurrent capecitabine, either before or after receiving additional chemotherapy. Institutionally, chemoradiation was favored first however patients with para-aortic nodal involvement were favored to start with chemotherapy initially or medical oncologists’ preference. The decision to pursue surgery was dependent on clinical response following TNT. Patients were excluded if they had squamous cell carcinoma or other histology, had received prior radiation therapy for rectal adenocarcinoma, or were medically inoperable due to co-morbidities.

Treatment response was assessed on MRI and endoscopy between 1 and 6 months after RT completion. Digital rectal exam was performed every 3 months and MRI pelvis, flexible sigmoidoscopy, abdominal CT imaging every 6 months. Colonoscopy was performed at 1, 3 and 5 years. The median time from treatment start to first tumor response assessment on either MRI or endoscopy, whichever occurred first, was 10.9 weeks (range, 8.7 – 106.0 weeks). For patients who underwent chemotherapy first, the median time to first tumor response assessment was 31.9 weeks (range, 27.4 – 106.0 weeks). For patients who underwent CRT first, the median time to first tumor response assessment was 10.1 weeks (range, 8.7 – 39.6 weeks). Patients were categorized into cCR or incomplete response. Complete response was defined as no observable residual tumor or evidence of lymph node involvement on MRI and no evidence of residual tumor on endoscopy. Patients with no evidence of disease on endoscopy at 24 weeks after completion of CRT were considered clinical complete responders. All near-complete responders converted to complete responders by 24 weeks.

Kaplan-Meier method was utilized to estimate the 1- and 2-year surgery- and permanent ostomy-free survivals. Cox regression analysis evaluated associations between surgery- and permanent ostomy-free survivals and various factors of interest, including patient and tumor characteristics and clinical response. We used Chi-squared test to compare patients who received CRT followed by consolidation chemotherapy to those who received induction chemotherapy followed by CRT in regard to associations with cCR and surgery free survival.

Lymphopenia and markers of systemic inflammation have been associated with overall survival in rectal cancer (11, 12). We analyzed absolute lymphocyte counts (ALC), neutrophil to lymphocyte ratios (NLR), and platelet to lymphocyte ratios (PLR) at baseline prior to CRT and at 3-month intervals following CRT. A Chi-squared analysis compared ALC, NLR, and PLR at all timepoints to rates of cCR and surgery.

## Results

### Patient, tumor, and treatment characteristics

Between 2013 and 2022, a total of 45 patients treated with TNT for stage II-IV rectal adenocarcinoma at our institution were identified. Patient demographics, disease characteristics and treatment parameters are outlined in **Tables 1**, **2**. The median age at diagnosis was 60 years (range, 50 – 68) and 64.4% of patients were male. Five patients (11.1%) had a history of prior cancer, excluding non-melanoma skin cancer. Of these with a history of cancer, three had prostate cancer, one had breast cancer, and one had melanoma. One patient with a history of prostate cancer had undergone brachytherapy, but otherwise these patients did not have a history of prior XRT. Half (51.1%) of patients were former or current tobacco users and 24.4% had a history of diabetes. At diagnosis, 93.3% (n=42) had an Eastern Cooperation Oncology Group (ECOG) performance status of 0 or 1.

**Table 1:** Summary of clinical, imaging, and disease characteristics.

Characteristic	*n* or median	% or IQR
Age at diagnosis, years	60	50 - 68
Male sex	29	64.4
History of previous cancer^1^	5	11.1
History of prior RT	1	2.2
Smoking history		
Never	22	48.9
Former tobacco use	16	35.6
Current tobacco us	7	15.6
Diabetes	11	24.4
ECOG		
0	29	64.4
1	13	28.9
2	2	4.4
3	1	2.2
Primary tumor size, cm	4.7	2.5 – 11.0
Distance from anal verge, cm	4.1	0.0 – 12.0
< 5 cm	25	55.6
5 cm ≤ x < 10 cm	16	35.6
≥ 10 cm	3	6.7
CEA at diagnosis	3.0	0 - 80
Baseline MRI*		
Extramural vascular Invasion	4	11
MRF threatening	13	36.1
Lateral lymph nodes present	11	28.2
Degree (%) of circumferential bowel invasion	67	50.0 – 100.0
T category		
2	2	4.4
3	36	80.0
4a	4	8.9
4b	3	6.7
N category		
0	21	46.7
1	18	40.0
2	6	13.3
Stage group		
IIA	18	40.0
IIB	1	2.2
IIC	2	4.4
IIIA	1	2.2
IIIB	15	33.3
IIIC	7	15.6
IVA	1	2.2

^1^Excluding non-melanoma skin cancer.

MRF, mesorectal fascia.

IQR, interquartile range.

*patients with evaluable MRI data.

**Table 2:** Summary of treatment parameters.

Variable	*n* or median	% or range
Radiation therapy		
Dose, Gy	54	50 – 66
Fractions	30	27 – 30
Treatment duration, days	42	34 – 54
Completed RT	45	100
Chemotherapy		
CNCT^1^	36	80
INCT^2^	9	20
Completed chemotherapy	34	75.6

^1^Consolidation chemotherapy.

^2^Induction chemotherapy.

Patients had stage II-IVA disease according to the Eighth Edition AJCC Cancer Staging Manual, most commonly stage IIA (40.0%), followed by IIIB (33.3%). Only one patient had stage IVA disease due to metastatic spread to low peri-aortic lymph node involvement, but was treated with definitive intent in absence of non-nodal distant metastasis. Primary tumor size defined on MRI was a median of 4.7 cm at its largest dimension (range, 2.5 – 11.0) with a median distance from the anal verge of 4.1 cm (range, 0.0 – 12.0). Fifty-six percent of primary tumors were located < 5 cm from the anal verge, 35.6% were between 5 cm and 10 cm, and 6.7% were 10 cm or further from the anal verge.

All patients underwent TNT consisting of XRT with concurrent capecitabine, preceded or followed by chemotherapy alone, with intent for non-operative management if cCR was reached. Eighty percent of patients received CRT first, and 20% received chemotherapy first. Four patients (8.9%) underwent diverting colostomy prior to CRT. The median dose of radiation received was 54 Gy in 30 fractions, consisting of 45 Gy elective nodal radiation with a sequential 9 Gy in 5 fraction primary tumor boost. All patients completed CRT. Patients were prescribed capecitabine twice daily on days of radiation. The most common chemotherapy regimens were XELOX (capecitabine/oxaliplatin), received by 53.3% of patients, or FOLFOX (fluorouracil/oxaliplatin/leucovorin), received by 31.1% of patients (**Table 3**). There was an average of 16 weeks of chemotherapy completed, and 75.6% of patients completed all planned cycles of chemotherapy. There were only 4 acute grade 3 toxicities, 2 in the CRT upfront arm and 2 in the chemo upfront arm; there were no grade 4+ acute toxicities.

**Table 3:** Summary of chemotherapy regimens.

Chemotherapy	*n*	%
XELOX	24	53.3
FOLFOX	14	31.1
XELOX/FOLFOX	3	6.7
FOLFIRINOX/FOLFIRI	1	2.2
FOLFOX/Pembrolizumab	1	2.2
Capecitabine	2	4.4

### Treatment response and outcomes following TNT

Of the 45 patients treated with TNT for rectal adenocarcinoma, 64.4% (n=29) reached cCR on MRI or endoscopy at 6 months after completing TNT (**Table 4**). Of the 29 patients with cCR, all but one were initially managed without surgery. This patient had partial treatment response on pathology following LAR, with pathology revealing no gross tumor but microscopic residual cancer cells with moderate treatment effect. This case was staged as pNX as no lymph nodes were identified on gross or histologic examination. Three additional patients who had cCR developed tumor regrowth (10.3%) and underwent salvage surgery, two of whom required a permanent ostomy. None of these had concomitant distant failure. One patient who had cCR developed distant metastasis. All but one patient with cCR, including all who experienced tumor regrowth, were alive at last time of follow up. The patient’s cause of death was not specified in the medical record, however, in the setting of multiple other medical comorbidities, they had elected to discontinue rectal cancer surveillance after two years.

**Table 4:** Treatment response and outcomes following TNT.

	LAR^1^ n (%)	APR^1^ n (%)	No TME^2^ n (%)	Total n (%)
**All patients (n=45)**	8 (17.8)	5 (11.1)	32 (71.1)	45 (100.0)
cCR	1 (2.2)	0 (0.0)	28 (62.2)	29 (64.4)
Permanent ostomy^3^	1 (2.2)	5 (11.1)	4 (8.9)	10 (22.2)
Local recurrence or regrowth	1 (2.2)	1 (2.2)	7 (15.6)	9 (20.0)
Salvage surgery	1 (2.2)	0 (0.0)	5 (11.1)	6 (13.3)
Distant metastasis	2 (4.4)	0 (0.0)	3 (6.7)	5 (11.1)
With concomitant local failure	0 (0.0)	0 (0.0)	2 (4.4)	2 (4.4)
Alive	6 (13.3)	5 (11.1)	31 (68.9)	42 (93.3)
**cCR (n=29)**	1 (3.4)	0 (0.0)	28 (96.6)	29 (100.0)
Permanent ostomy^3^	0 (0.0)	0 (0.0)	2 (6.9)	2 (6.9)
Tumor regrowth	0 (0.0)	0 (0.0)	3 (10.3)	3 (10.3)
Salvage surgery	0 (0.0)	0 (0.0)	3 (10.3)	3 (10.3)
Distant metastasis	0 (0.0)	0 (0.0)	1 (3.4)	1 (3.4)
With concomitant local failure	0 (0.0)	0 (0.0)	0 (0.0)	0 (0.0)
Alive	1 (3.4)	0 (0.0)	27 (93.1)	28 (96.6)
**Incomplete response (n=16)**	7 (43.8)	5 (31.6)	4 (25.0)	16 (100.0)
Permanent ostomy^3^	1 (6.3)	5 (31.6)	2 (12.5)	8 (50.0)
Local recurrence/progression	1 (6.3)	1 (6.3)	4 (25.0)	6 (37.5)
Salvage surgery	1 (6.3)	0 (0.0)	2 (12.5)	3 (18.8)
Distant metastasis	2 (12.5)	0 (0.0)	2 (12.5)	4 (25.0)
With concomitant local failure	0 (0.0)	0 (0.0)	2 (12.5)	2 (12.5)
Alive	5 (31.6)	5 (31.6)	4 (25.0)	14 (87.5)

^1^Excluding surgery for regrowth.

^2^Total mesorectal excision, excluding surgery for regrowth.

^3^Including those created in salvage surgery.

LAR, low anterior resection; APR, Abdominoperineal resection; TME, total mesorectal excision.

Sixteen patients (35.6%) had incomplete clinical response following TNT (**Table 4**). A total of 12 patients underwent surgical resection of their tumor, none of which had pathologic complete response, and 4 patients declined surgical resection. Eight patients initially with incomplete response ultimately had a permanent ostomy. Six of these had an APR upfront, two initially declined surgery but later underwent APR for progression of disease, and one patient who initially had a LAR later underwent APR for recurrence. In addition to this one patient who had an APR for recurrence, another patient who had incomplete response after TNT and initially had an APR later developed a recurrence, which was unresectable and was treated with chemotherapy alone. Four patients who initially had incomplete clinical response developed distant metastasis, two of whom had originally declined surgery. Fourteen of 16 patients (87.5%) with incomplete clinical response were alive at last time of follow up.

**Figure 1:**
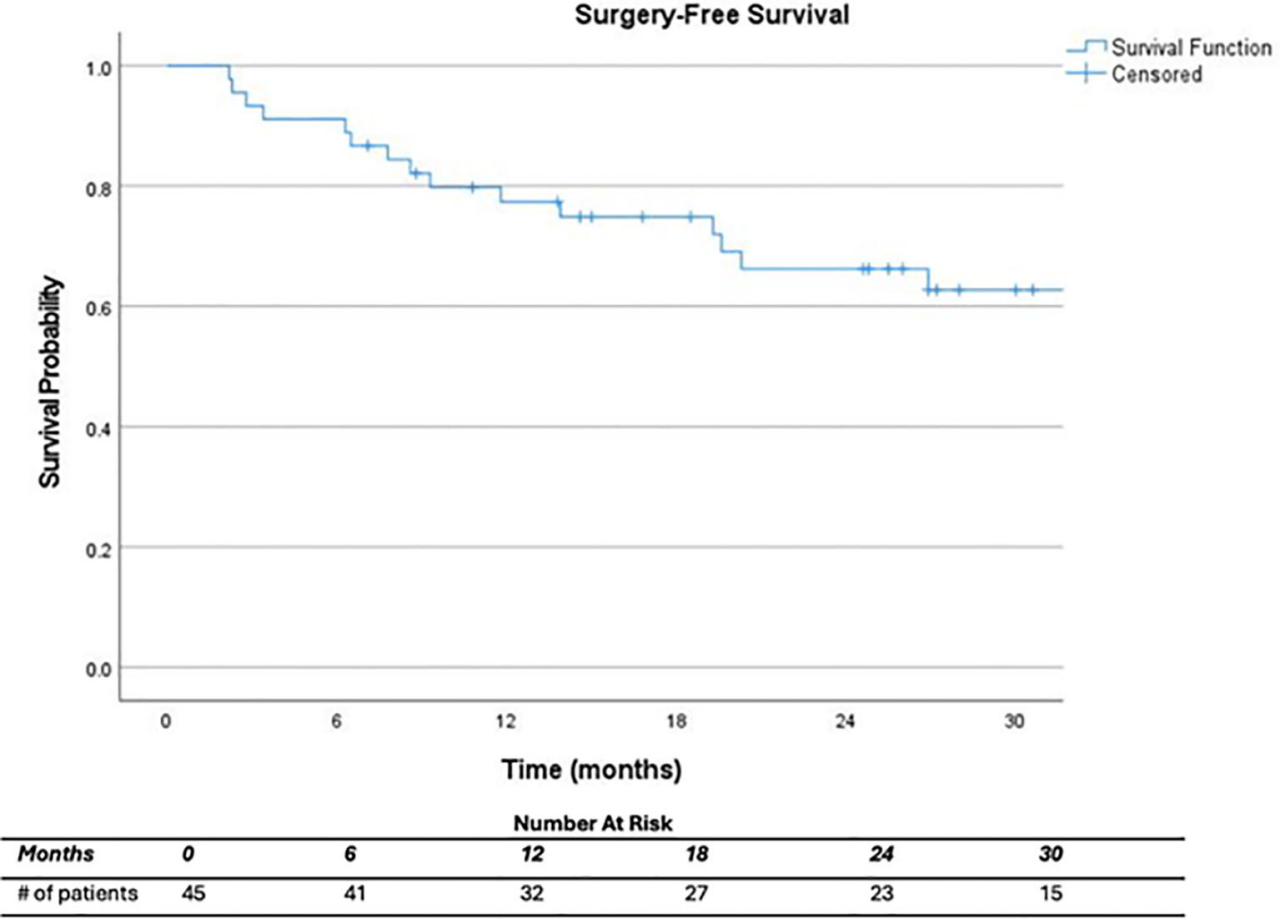
Surgery-free survival.

**Figure 2:**
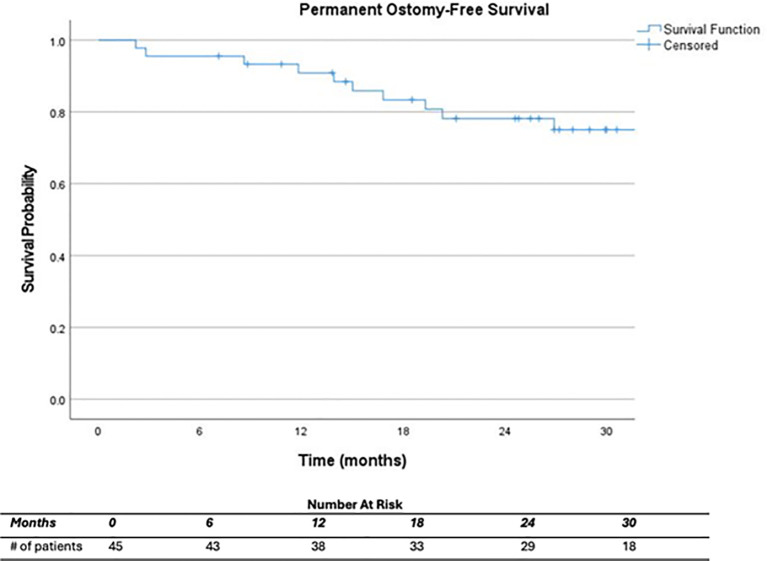
Permanent ostomy-free survival.

At a median follow up of 32.0 months (range, 7.1 – 86.1) after completion of CRT, 22.2% (n=10) of all patients had a permanent colostomy, half of which were completed during salvage surgery following local recurrence. For those with a cCR, 2 patients required permanent ostomy for tumor regrowth. For those who did not achieve a cCR, 5 required an ostomy at initial surgery, 2 patients who initially declined surgery later had an APR for progression of disease, and 1 patient who underwent LAR and ostomy reversal later underwent an APR with end colostomy for locally recurrent disease (**Table 4**). At one and two years, respectively, surgery-free survival was 77.3% and 66.2%, and permanent ostomy-free survival was 90.9% and 78.2% (**Figures 1**, **2**). On Cox regression model, cCR on 6 month post-CRT endoscopy was associated with surgery-free survival (p=0.006) and permanent ostomy-free survival (p=0.033). Clinical response at 1 and 3 months did not predict surgery-free survival nor permanent ostomy-free survival.

### Association with sequence of TNT modalities

**Figure 3:**
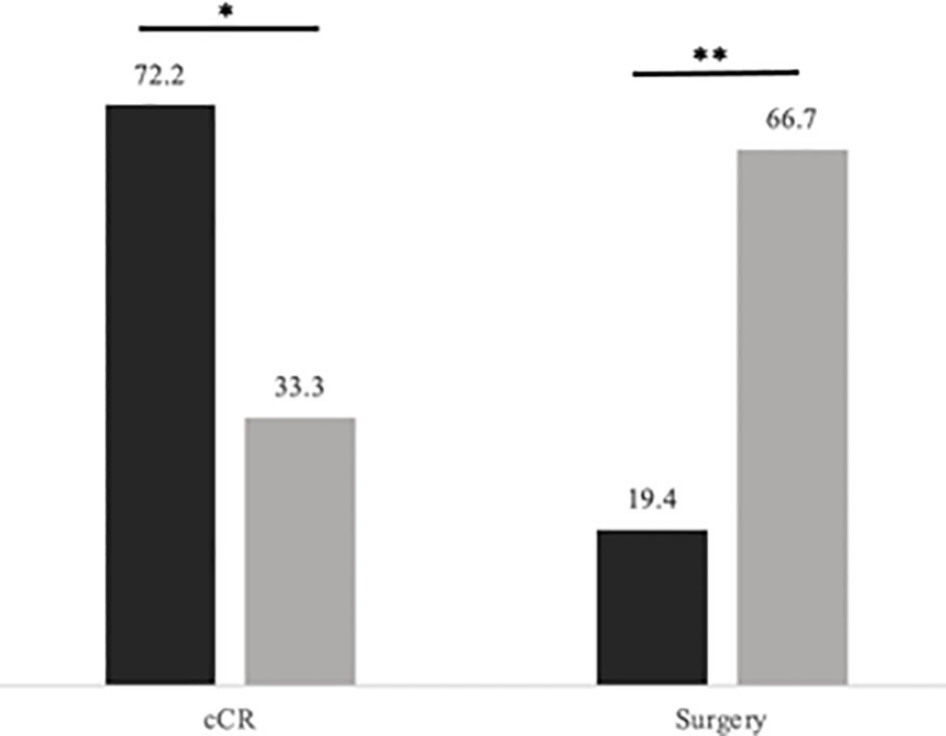
Rates of cCR and surgery by TNT sequence. Levels of significance: *< 0.05 **< 0.01. Black represents CRT first followed by consolidation chemotherapy; Gray represents induction chemotherapy followed by CRT. Rates (in percentage of patients) of cCR and surgery by TNT sequence.

**Figure 4:**
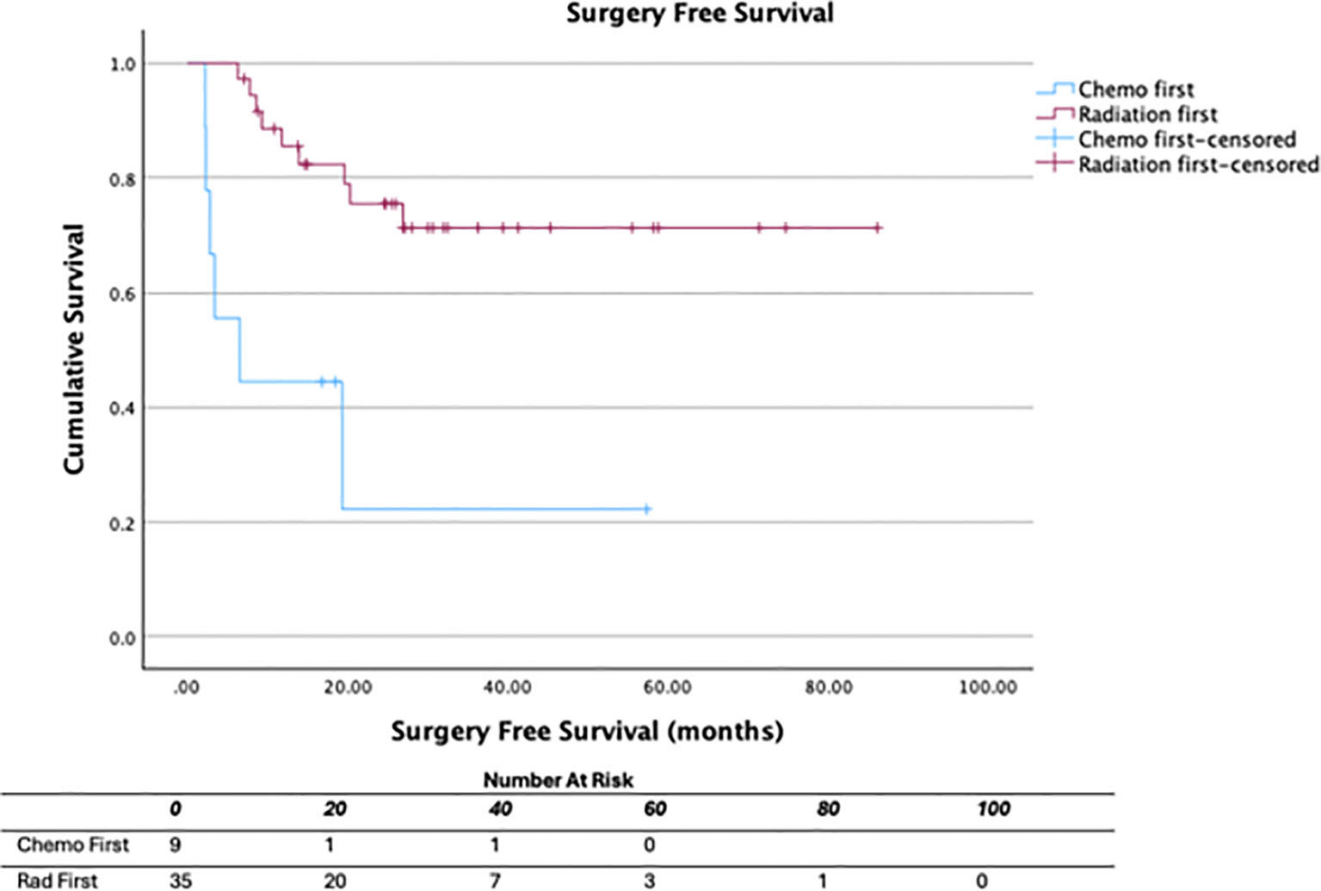
Surgery-free survival by TNT sequence.

We additionally compared patients who had chemotherapy first to those who completed CRT first (**Table 5**). Patients who completed CRT first followed by consolidation chemotherapy were significantly more likely to reach cCR and less likely to undergo surgery compared to those who received chemotherapy first (**Figures 3**, **4**). 33.3% of patients who received chemotherapy first had cCR compared to 72.2% of patients who received CRT first (p=0.029). 66.7% of patients who received chemotherapy first eventually underwent surgery, compared to 19.4% who received CRT first (p=0.005), with permanent ostomy rates of 44.4% versus 16.7% in the chemotherapy versus CRT first groups respectively.

**Table 5:** Characteristics of patients by TNT sequence.

Characteristic	CNCT^1^	INCT^2^
Age at diagnosis, years, median (range)	60 (50 - 69.5)	56 (46.5 - 65.5)
Male sex, n (%)	23 (51.1)	6 (66.7)
History of previous cancer^3^, n (%)	4 (11.1)	1 (11.1)
History of prior RT, n (%)	1 (2.8)	0 (0.0)
Smoking history, n (%)		
Never	17 (47.2)	4 (44.4)
Former tobacco use	13 (36.1)	2 (22.2)
Current tobacco use	4 (11.1)	3 (33.3)
Diabetes, n (%)	7 (19.4)	2 (22.2)
ECOG, n (%)		
0	25 (69.4)	4 (44.4)
1	9 (25.0)	4 (44.4)
2	2 (5.6)	0 (0.0)
3	0 (0.0)	1 (11.1)
Primary tumor size, cm, median	4.9 (3.8 - 6.0)	4.7 (3.7 - 6.9)
Distance from anal verge, cm, median	4.1 (2.0 - 7.0)	4.3 (2.0 - 6.8)
CEA at diagnosis, median (range)	3.3 (1.4 - 7.6)	3.1 (1.3 - 5.6)
T category, n (%)		
2	2 (5.6)	0 (0.0)
3	28 (77.8)	8 (88.9)
4a	4 (11.1)	0 (0.0)
4b	2 (5.6)	1 (11.1)
N category, n (%)		
0	14 (38.9)	6 (66.7)
1	17 (47.2)	2 (22.2)
2	5 (13.9)	1 (11.1)
Stage group, n (%)		
IIA	13 (36.1)	5 (55.6)
IIB	1 (2.8)	0 (0.0)
IIC	1 (2.8)	1 (11.1)
IIIA	1 (2.8)	0 (0.0)
IIIB	14 (38.9)	1 (11.1)
IIIC	6 (16.7)	1 (11.1)
IVA	0 (0.0)	1 (11.1)

^1^Consolidation chemotherapy.

^2^Induction chemotherapy.

^3^Excluding non-melanoma skin cancer.

### Association with ALC, NLR, and PLR on rates of cCR and surgery

On Chi-squared analysis, 3-month post-CRT PLR was associated with a statistically significant difference in rate of cCR. There was no significant association with rates of cCR or surgery at other timepoints or for ALC or NLR at any timepoints.

## Discussion

Treatment paradigms for low-lying locally advanced rectal adenocarcinoma have evolved in recent years toward earlier radiation and systemic therapy and non-operative management in select patients. The implementation of TNT yielded improved outcomes for patients with rectal cancer. Garcia-Alguilar et al. demonstrated that implementing a regimen of chemotherapy after CRT increased rates of pathological complete response at the time of surgery (14). The PRODIGE 23 trial showed that neoadjuvant chemotherapy added to neoadjuvant CRT improved disease-free survival and decreased toxicity compared to adjuvant chemotherapy (15). Transitioning to TNT not only improved treatment response, but potentially allows patients to pursue non-operative treatment (8, 15). Results from the International Watch & Wait Database show a 2-year incidence of local regrowth of 25.2% and 5-year disease-specific survival of 94% in patients with a cCR after TNT without surgery (9). These evolving approaches have achieved substantial advances in organ sparing and high disease-free survival. In patients with microsatellite instability, neoadjuvant immunotherapy upfront represents another exciting option for treatment de-escalation and organ-sparing (16).

Our institutional results are in line with historic observations, which support the use of TNT with a non-operative approach for rectal adenocarcinoma. Overall, patients treated with TNT with non-operative intent at our institution demonstrated a 77.3% and 66.2% surgery-free survival at 1- and 2-years, respectively. In comparison, the OPRA trial reported 3-year TME-free survival ranging between 41% and 53% depending on TNT sequence (8). These differences may in part be attributable to a greater number of patients at our institution who declined surgery and/or a great number treated with CRT first compared to those included in the OPRA study. Of those who reached cCR in our study, 86.2% had no local or distant recurrence, compared to 74.2% in the analysis of the International Watch & Wait Database conducted by van der Valk et al. (9).

Results of our study revealed an advantage to completing CRT prior to chemotherapy in the sequence of TNT. Significantly more patients who received CRT first reached cCR; in addition fewer CRT first patients underwent surgery or required a permanent ostomy. This observation corroborates that of the OPRA trial which showed that initiating TNT with CRT may be associated with higher rates of organ preservation (8). These data indicate that the sequence of treatment in TNT has clinically significant impact on outcomes, favoring the completion of CRT prior to chemotherapy.

There is an ongoing discussion regarding the implementation of short-course compared to long-course XRT. While the RAPIDO trial showed that short-course radiation as a part of TNT improved distant metastasis and disease-related treatment failure compared to the standard treatment of chemoradiation followed by surgery and adjuvant chemotherapy (5), the more recent publication on locoregional recurrence reports possibly inferior local recurrence in this short-course radiation group (6). In our study, long course XRT was used for all patients as the Trans-Tasman Radiation Oncology Group Trial suggested potentially better local control with long-course XRT compared to short-course, particularly for distal tumors, which comprise a large proportion of patients in this study and are at high risk of receiving a permanent ostomy (17).

Appropriate selection of patients for the watch-and-wait protocol is a fundamental investigative question to maximize our patients’ outcomes. Biomarkers of treatment response and recurrence are exciting avenues that might improve patient outcomes. In this study, platelet to lymphocyte ratios (PLR) at 3 months after completion of CRT was associated with rates of cCR. PLR is thought to be a marker of systemic inflammation, and elevated PLR has been correlated with poor overall survival and progression-free survival in a variety of cancers (11). A recent meta-analysis of PLR in colorectal cancer has shown PLR to correlate with overall survival, recurrence, and such poor clinical features as poor tumor differentiation and depth of infiltration (12). While this meta-analysis primarily looked at pretreatment PLR, the importance of post-treatment PLR remains unclear. In addition to these simple blood assessments, a new age of liquid biopsies to assess circulating tumor cells and circulating tumor DNA is providing new avenues to evaluate continued treatment efficacy and to provide early clues and opportunities to identify patients needing treatment escalation and to manage recurrent or metastatic disease (18, 19). A current institutional protocol is incorporating these biomarkers in TNT assessment.

Another critical clinical question is the timing of close follow up after TNT when implementing the non-operative approach. In our study, clinical response on endoscopy at 6 months was also associated with improved surgery- and ostomy-free survival rates, but not at earlier timepoints. This result suggests continued tumor regression after one month, which is consistent with NCCN guidelines to restage rectal cancer patients after at least 8 weeks following XRT (20). In our study, time to first response assessment on either MRI or endoscopy was completed a median of 10.9 weeks after start of TNT with continued response assessment at later timepoints prior to decision to pursue surgery or organ preservation based on cCR at 24 weeks (6 months). While this timeline is consistent with practice guidelines, there is substantial variability in tumor restaging schedules in prior studies. International consensus guidelines published in 2021 recommend the optimal timing of determination of cCR as 24 weeks after the start of TNT, or 34–38 weeks after the start of TNT with prolonged consolidation chemotherapy (21). Sandwich TNT, recently reported in a retrospective study (22), may be another option for a watchful waiting approach with promising cCR/partial radiological clinical response rates as high as 70% only 7 weeks after TNT.

Our current limitations for this study include its retrospective nature, sample size of 45, and representation of a single-institution experience. In addition, the percentage of patients treated with chemotherapy upfront was much smaller than those treated with upfront CRT and patients were not randomized to either TNT sequence. However, despite the small number of patients in the upfront chemotherapy group, there was still a significant decrease in their cCR rate, a finding supported by the OPRA trial (8).

## Conclusions

Our institutional results support the use of TNT in select patients for definitive treatment of rectal adenocarcinoma with good rates of organ preservation and surgery-free survival. The order of treatment modalities in TNT was significant in rates of cCR and surgery, favoring a CRT-first approach. Those who underwent CRT before chemotherapy were more likely to have a cCR and were less likely to have surgery. Further studies aimed to identify predictors of which patients are likely to achieve cCR as well as the interplay between sequencing of TNT and timing of follow up surveillance assessment are warranted.

## Data availability statement

The raw data supporting the conclusions of this article will be made available by the authors, without undue reservation.

## Ethics statement

The studies involving humans were approved by University of Virginia Institutional Review Board. The studies were conducted in accordance with the local legislation and institutional requirements. The ethics committee/institutional review board waived the requirement of written informed consent for participation from the participants or the participants’ legal guardians/next of kin because the nature of the retrospective study design in patients provided standard of care treatment and did not provide an opportunity for consent but a waiver was obtained.

## Author contributions

EA: Conceptualization, Data curation, Formal analysis, Investigation, Methodology, Supervision, Validation, Writing – review & editing, Project administration. EV: Data curation, Formal analysis, Investigation, Writing – original draft. HB: Formal analysis, Methodology, Validation, Writing – review & editing, Software, Visualization. JS: Writing – review & editing. PK: Writing – review & editing. TH: Writing – review & editing. SH: Writing – review & editing. MR: Writing – review & editing. TL: Writing – review & editing. CF: Writing – review & editing. E-MJ: Conceptualization, Formal analysis, Investigation, Methodology, Project administration, Resources, Supervision, Validation, Writing – review & editing.
